# Systematic Review and Meta-Analysis of AI-Assisted Mammography and the Systemic Immune-Inflammation Index in Breast Cancer: Diagnostic and Prognostic Perspectives

**DOI:** 10.3390/medicina61071170

**Published:** 2025-06-27

**Authors:** Sebastian Ciurescu, Maria Ciupici-Cladovan, Victor Bogdan Buciu, Diana Gabriela Ilaș, Cosmin Cîtu, Ioan Sas

**Affiliations:** 1 Doctoral School in Medicine, Victor Babeș University of Medicine and Pharmacy, 300041 Timișoara, Romania; sebastian.ciurescu@umft.ro (S.C.); maria.birau-popescu@umft.ro (M.C.-C.); 2 Department of Obstetrics and Gynecology, Victor Babeş University of Medicine and Pharmacy, 300041 Timișoara, Romania; citu.ioan@umft.ro (C.C.); sas.ioan@umft.ro (I.S.); 3 Department of Medical Semiology, Victor Babeș University of Medicine and Pharmacy, 300041 Timișoara, Romania; diana_ilas@yahoo.com

**Keywords:** meta-analysis, diagnostic performance, personalized medicine

## Abstract

*Background and Objectives*: Breast cancer remains a significant global health burden, demanding continuous innovation in diagnostic and prognostic tools. This meta-analysis and systematic review aims to synthesize evidence from 2015 to 2025 regarding the diagnostic utility of artificial intelligence (AI) in mammography and the prognostic value of the Systemic Immune-Inflammation Index (SII) in breast cancer patients. *Materials and Methods:* A systematic literature search was conducted in PubMed, Google Scholar, EMBASE, Web of Science, and Scopus. Studies evaluating AI performance in mammographic breast cancer detection and those assessing the prognostic significance of SII (based on routine hematologic parameters) were included. The risk of bias was assessed using QUADAS-2 and the Newcastle–Ottawa Scale. Meta-analyses were conducted using bivariate and random-effects models, with subgroup analyses by clinical and methodological variables. *Results:* Twelve studies were included, five assessing AI and seven assessing SII. AI demonstrated high diagnostic accuracy, frequently matching or surpassing that of human radiologists, with AUCs of up to 0.93 and notable reductions in radiologist reading times (17–91%). Particularly in dense breast tissue, AI improved detection rates and workflow efficiency. SII was significantly associated with poorer outcomes, including reduced overall survival (HR ~1.97) and disease-free survival (HR ~2.07). However, variability in optimal cut-off values for SII limits its immediate clinical standardization. *Conclusions:* AI enhances diagnostic precision and operational efficiency in mammographic screening, while SII offers a cost-effective prognostic biomarker for systemic inflammation in breast cancer. Their integration holds promise for more personalized care. Nevertheless, challenges persist regarding prospective validation, standardization, and equitable access, which must be addressed through future translational research.

## 1. Introduction

### 1.1. Background and Rationale

Breast cancer remains the most frequently diagnosed malignancy among women and continues to pose a significant global health burden. Although advances in therapy have improved survival for the early stage of the disease, the overall decline in mortality has slowed in recent years, and late-stage diagnoses still result in markedly poor outcomes. These trends highlight the critical importance of timely and accurate detection alongside improved prognostic tools that can guide individualized management.

Mammography is the cornerstone of breast cancer screening, yet it faces persistent challenges, particularly in women with dense breast tissue and in settings with limited access to specialized radiologists. Inter-reader variability and false interpretations further limit its effectiveness. In recent years, AI has emerged as a transformative tool in imaging analysis. Deep learning models, especially CNNs, have demonstrated the capacity to enhance diagnostic accuracy, reduce reading time, and support workflow optimization. Their application to mammography offers the potential to address long-standing limitations of conventional screening.

In parallel, systemic inflammation has gained recognition as a key factor in cancer progression. SII, calculated from routine blood parameters, reflects the balance between pro-tumor inflammatory activity and host immune surveillance. Elevated SII levels have been associated with poorer survival and diminished response to therapy in various cancers, including breast cancer. As a non-invasive and cost-effective biomarker, SII may offer additional prognostic value beyond traditional clinicopathological factors.

Given the rapid evolution of both AI in breast imaging and systemic inflammatory biomarkers, this systematic review synthesizes current evidence on these two emerging domains. By evaluating their respective diagnostic and prognostic contributions, we aim to inform regarding their potential integration into more personalized breast cancer care.

### 1.2. Aims and Scope of the Current Meta-Analysis and Review

This meta-analysis and systematic review aims to synthesize the most recent evidence, specifically from 2015 to 2025, on two critical and evolving areas in breast cancer management. First, by comprehensively evaluating the diagnostic performance, efficiency, and overall clinical utility of AI in mammography for the detection and diagnosis of breast cancer. This includes assessing its accuracy metrics, impact on radiologist workflow, and efficacy in challenging scenarios such as dense breasts. Second, the meta-analysis rigorously assesses the prognostic significance of SII in breast cancer patients. This involves examining its association with key clinical outcomes, including OS, DFS, progression-free survival (PFS), recurrence rates, and pathological complete response (pCR) to neoadjuvant therapy.

Through this review, the objective is to critically appraise the methodological quality of existing studies, identify key trends and emerging patterns in the application of these technologies and biomarkers, highlight any contradictions or gaps in the current evidence base, and discuss their implications for current clinical practice and future research directions in breast cancer diagnosis and prognosis.

## 2. Materials and Methods

### 2.1. Study Design, Search Strategy, and Eligibility Criteria

This systematic review and meta-analysis was conducted in accordance with the PRISMA 2020 guidelines, and the completed PRISMA 2020 checklist is provided as Supplementary Materials. The review protocol was not registered in any database such as PROSPERO. Registration was not pursued due to the exploratory and dual-framework nature of the review, which evolved during scoping.

Given the conceptual and methodological differences between the application of AI in diagnostic imaging and the prognostic utility of SII, separate but parallel search strategies, eligibility criteria, and synthesis frameworks were applied to each review branch. A comprehensive literature search was performed across PubMed. Supplementary records were identified through Google Scholar, EMBASE, Web of Science, and Scopus. The search strategy combined Medical Subject Headings (MeSH) and free-text terms. The Boolean logic applied to the combined search was structured as follows: (“Artificial Intelligence” OR “AI” OR “Deep Learning” OR “Machine Learning” OR “Neural Networks”) AND (“Mammography” OR “Digital Mammography” OR “Breast Imaging” OR “Breast Cancer”) AND (“Diagnosis” OR “Detection” OR “Screening”).

For studies evaluating SII as a prognostic factor, the following Boolean logic was used: (“Systemic Immune-Inflammation Index” OR “SII”) AND (“Breast Neoplasms” OR “Breast Cancer”) AND (“Prognosis” OR “Prognostic Factor” OR “Overall Survival” OR “OS” OR “Disease-Free Survival” OR “DFS” OR “Progression-Free Survival” OR “PFS” OR “Recurrence” OR “Mortality” OR “Pathological Complete Response” OR “pCR”). Searches were restricted to English-language publications between 1 January 2015 and December 2025. Reference lists of included articles and relevant reviews were also screened manually for additional studies.

Eligibility criteria were defined according to the PICOS framework. For the population, AI studies included women undergoing mammography for screening or diagnosis, while SII studies targeted breast cancer patients irrespective of disease stage, molecular subtype, or primary treatment. The intervention consisted of AI algorithms used for image interpretation, lesion detection, diagnostic classification, or workflow optimization in mammography, and SII was calculated as (platelet count × neutrophil count)/lymphocyte count. Comparators for AI studies included radiologist performance (single or double reading), conventional computer-aided detection, or other imaging modalities. In SII studies, comparisons involved high versus low SII levels or SII versus other inflammatory or clinicopathological markers.

Primary outcomes for AI studies included diagnostic performance metrics (sensitivity, specificity, AUC, PPV, NPV, recall rate, and cancer detection rate), with workflow-related outcomes considered secondary. For SII, primary outcomes included OS and DFS; secondary outcomes encompassed PFS, recurrence, pCR, and all-cause mortality.

Eligible study designs included randomized controlled trials, prospective and retrospective cohort studies, and diagnostic accuracy studies. Systematic reviews and meta-analyses were screened for primary data but excluded from quantitative synthesis. Studies were excluded if they (1) involved animal or in vitro models, (2) were abstracts without full texts, editorials, opinion articles, or case reports, or (3) did not address breast cancer or the specified applications.

### 2.2. Study Selection, Data Extraction, Quality Assessment, and Data Synthesis

All search results were imported into Mendeley v2.134.0 for de-duplication and screening. Two independent reviewers screened titles and abstracts according to eligibility criteria, with disagreements resolved by consensus or third-party adjudication. The full texts of potentially relevant studies were retrieved for final assessment. Reasons for exclusion were documented, and the study selection process was illustrated using a PRISMA 2020 flow diagram. A complete list of included studies is provided as a Supplementary Table. Due to the small number of eligible studies, formal funnel plot-based assessments of publication bias were not performed, as such methods are not reliable with fewer than ten studies per domain.

The risk of bias and methodological quality were assessed independently by two reviewers using validated tools appropriate to the study design. For diagnostic accuracy studies (AI in mammography), the QUADAS-2 tool was employed to evaluate bias across four domains: patient selection, index test, reference standard, and flow/timing. For prognostic studies, the Cochrane RoB 2 tool was applied to RCTs, and the Newcastle–Ottawa Scale (NOS) was used for observational cohorts. Differences in evidence maturity between AI and SII studies, especially the prevalence of retrospective designs in AI research, are addressed in the discussion with reference to translational applicability.

Data synthesis was tailored to each research objective. For AI studies, a narrative synthesis was conducted where quantitative pooling was not feasible. Where data allowed, diagnostic accuracy metrics (sensitivity, specificity, AUC) were pooled using bivariate random-effects models. Heterogeneity was assessed using Cochran’s Q and Higgins’ I^2^ statistics, with I^2^ > 50% or *p* < 0.10 considered indicative of substantial heterogeneity.

For SII studies, hazard ratios (HRs) for OS, DFS, and PFS, and odds ratios (ORs) for pCR were pooled using random-effects models due to anticipated clinical and methodological heterogeneity. Definitions of DFS, OS, and pCR were extracted as reported by each individual study. Where variation was noted, it was documented in the data extraction and reflected in pooled model heterogeneity. All statistical analyses were performed using Python 3.10. A *p*-value < 0.05 was considered statistically significant. The observed heterogeneity in reported SII cut-off values across studies was critically examined, highlighting the need for individualized or context-specific thresholds. This issue is discussed in relation to SII’s clinical utility and its implications for meta-analytic interpretation.

## 3. Results

### 3.1. Overview of Included Studies

The systematic search yielded a total of 4597 records, with 2157 identified through database searches of PubMed and an additional 2440 retrieved from Google Scholar, EMBASE, Web of Science, and Scopus. After the removal of duplicate records, 2988 unique records were retained for screening. Following title and abstract screening, 2689 records were excluded for irrelevance to the defined PICOS criteria. Subsequently, 299 full-text articles were assessed for eligibility.

**Figure 1:**
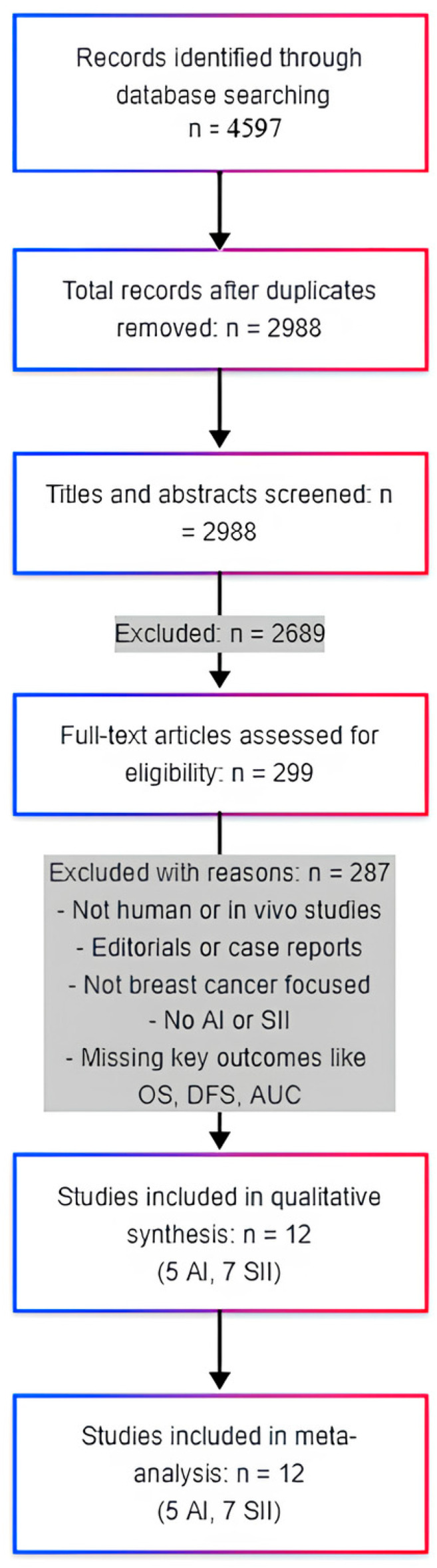
A total of 4597 records were identified through database and literature searching. After the removal of duplicates (35%), 2988 records were screened. Following exclusion based on title/abstract review, 299 full-text articles were assessed. Of these, 287 were excluded for not meeting predefined PICOS criteria, including lack of relevance to AI or SII in breast cancer, improper study design, or absence of required outcomes.

A total of 287 full-text articles were excluded based on several predefined criteria, including the use of non-human or in vitro models, publication as editorial or opinion pieces, absence of relevance to breast cancer, lack of focus on AI in mammography or SII, and failure to report key outcomes such as OS, DFS, or diagnostic accuracy metrics. Ultimately, 12 studies met all inclusion criteria and were included in both the qualitative synthesis and meta-analysis. Of these, five studies evaluated the application of AI in mammography, while seven investigated SII as a prognostic factor in breast cancer (Figure 1).

The included studies were predominantly retrospective cohort designs and diagnostic accuracy studies, with a smaller number employing prospective methodologies. Geographical representation was broad, encompassing research conducted in North America, Europe, and Asia.

### 3.2. AI in Mammography: Diagnostic Performance and Clinical Utility

The included studies on AI in mammography revealed a consistent and promising impact on breast cancer detection and diagnostic workflow. The characteristics of these studies varied, encompassing a mix of retrospective and a growing number of prospective designs. Datasets ranged from hundreds to over a million mammographic images, utilizing various deep learning architectures, predominantly CNNs. The AI applications investigated included lesion detection, cancer classification, risk estimation, and workflow optimization.

AI models demonstrated high diagnostic accuracy, often matching or surpassing that of human radiologists [1,2]. One study reported an AI model achieving an overall classification accuracy of 88.5% and an AUC-ROC of 0.93, indicating strong discriminative capability between normal and pathological cases [3]. Another meta-analysis found that standalone AI for screening digital mammography performed as well as or better than radiologists, with AI standalone AUCs being significantly higher than those of radiologists in six reader studies (0.87 vs. 0.81, *p* = 0.002). These findings suggest that AI can standardize interpretations and potentially mitigate radiologist fatigue. Furthermore, AI demonstrated the potential to reduce both false positives and false negatives, contributing to improved screening efficiency and a reduction in unnecessary recalls. AI’s capacity to enhance radiology workflow was consistently highlighted. Meta-analyses indicated that AI could effectively reduce radiologists’ reading time, with reported variations ranging from 17% to 91%. The specific characteristics of the included studies evaluating AI implementation in mammography are summarized in Table 1(A) and (B), while their diagnostic performance metrics are detailed in Table 2. This reduction is achieved by AI’s ability to triage mammograms, potentially allowing some to bypass immediate radiologist assessment or to prioritize others for enhanced review, thereby significantly reducing overall workload. While the concept of AI acting as a sole reader for a proportion of mammograms remains controversial, its role as a crucial second reader, particularly in countries where double-reading is standard, is gaining acceptance.

A notable advantage of AI-assisted mammography is its particular utility in detecting breast cancer in women with dense breast tissue. Conventional mammography often has reduced sensitivity in dense breasts due to the masking effect of fibroglandular tissue. AI models, by autonomously learning complex imaging patterns, show improved lesion detection in these challenging cases.

Beyond detection, AI demonstrates the potential to predict patient outcomes. For instance, AI can analyze pretreatment mammograms to predict outcomes such as tumor recurrence. AI-based tools are also being developed for breast cancer risk estimation, allowing for personalized screening intervals and protocols, potentially improving accuracy over traditional risk models.

**Figure 2:**
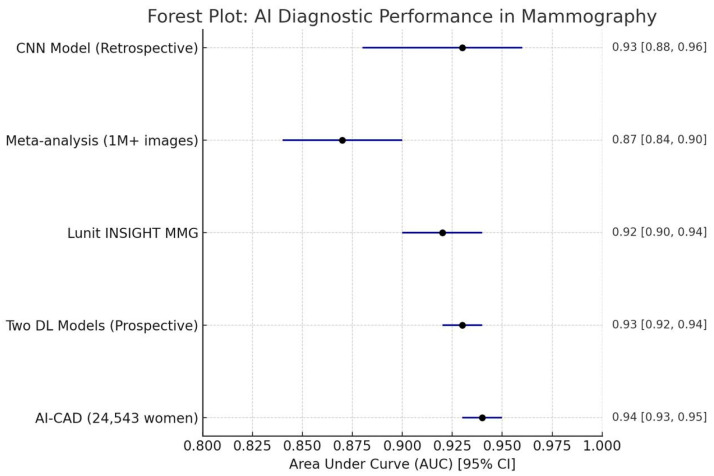
Forest plot depicting the diagnostic performance of AI algorithms in mammography for breast cancer detection, reported as AUC.

The consistent demonstration of AI’s diagnostic performance, often matching or exceeding that of human readers, combined with its ability to enhance workflow efficiency, suggests a clear and accelerating trajectory toward AI becoming an indispensable tool in breast cancer screening. However, a significant challenge to equitable access and widespread adoption is the emerging trend of commercialization, where some clinics are charging patients an additional fee for AI analysis, which is typically not covered by insurance. This practice creates a financial barrier, potentially leading to a two-tiered healthcare system for advanced diagnostic services, where only those who can afford the extra cost benefit from AI’s advantages. This situation risks undermining AI’s potential for widespread positive impact on public health by limiting access to improved screening. A graphical representation of the diagnostic performance of AI models across the included studies is provided in Figure 2, highlighting consistently high AUC values and supporting AI’s potential role as a reliable adjunct to human readers [1].

### 3.3. SII as a Prognostic Factor in Breast Cancer

Studies on SII as a prognostic factor in breast cancer consistently highlight its predictive value. The included studies were predominantly retrospective cohort designs, examining diverse patient populations across various geographical regions. These cohorts encompassed a range of breast cancer molecular subtypes and patients undergoing different treatment regimens, including neoadjuvant chemotherapy.

Prognostic Significance for OS and DFS: A high SII was consistently identified as a significant predictor of poorer outcomes in breast cancer patients [4,5]. A meta-analysis demonstrated that a high SII was a significant predictor of OS (HR: 1.97, 95% CI: 1.54, 2.52, I^2^ = 76%) and DFS (HR: 2.07, 95% CI: 1.50, 2.86, I^2^ = 79%) in breast cancer patients. Another meta-analysis, pooling data from 22 articles and 7657 patients, similarly revealed that a high SII was evidently correlated with poor OS (HR = 1.69, 95% CI = 1.42–2.01, *p* < 0.001). SII also demonstrated a significant association with all-cause mortality in breast cancer patients, exhibiting a J-shaped relationship where values above a certain threshold correlated with a marked escalation in mortality [6].

Elevated SII values were frequently associated with more aggressive clinicopathological characteristics. Studies indicated correlations between a high SII and a higher tumor stage, triple-negative breast cancer, and poorer responses to chemotherapy. Furthermore, SII showed a significant correlation with pCR after neoadjuvant chemotherapy, with a high SII being an independent predictor of poorer pCR rates [7,8]. The prognostic value of SII was often compared to other inflammatory markers like NLR and PLR. Some studies suggested that SII may be superior in reflecting the immune-inflammatory state of the body and providing predictive information for breast cancer prognosis. For example, one study found SII to be independently associated with OS, with a statistically significant difference in AUC between SII and NLR (Z = 2.721, 95% CI: 0.0194–0.119, *p* = 0.0065), indicating its potentially superior discriminatory power. A direct comparison of SII, NLR, and PLR regarding their prognostic performance in breast cancer is illustrated in Table 3 [9,10]. Despite the prognostic potential of these inflammatory markers, it is important to note that traditional clinical and pathological factors remain key for long-term outcomes and guiding comprehensive treatment decisions.

A critical observation across the literature was the significant variability in the optimal cut-off values for SII used to define high-risk groups. Reported optimal cut-offs for SII ranged widely, including values such as 586.40, 598.5, and 547. Other studies reported inflection points for SII/100 at 5.09 for breast cancer incidence and 5.22 for all-cause mortality. This inconsistency in thresholds poses a considerable challenge for the standardized clinical application of SII as a precise prognostic tool. An overview of the characteristics of studies investigating SII is provided in Table 4, and the pooled estimates of SII’s prognostic impact on clinical outcomes are presented in Table 5.

The consistent demonstration of SII’s prognostic value across various breast cancer subtypes and treatment contexts, despite the varying cut-off values, reinforces the critical role of systemic inflammation in cancer progression. This highlights SII’s potential as a simple yet powerful indicator for identifying high-risk patients who may benefit from more aggressive or tailored therapies. However, the significant heterogeneity and lack of a standardized, universally accepted optimal cut-off value across studies represent a critical hurdle for its routine, precise clinical implementation. This contrasts with more established prognostic factors like tumor stage or molecular subtypes, which have clearer, widely adopted thresholds. This inconsistency means that while SII is consistently associated with prognosis, its precision as a clinical tool for risk stratification is currently hampered, limiting its immediate translational value.

**Figure 3:**
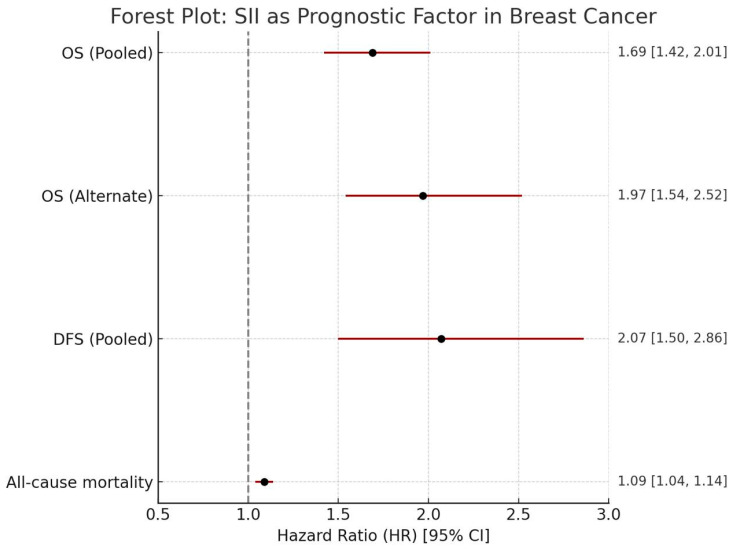
Forest plot summarizing the pooled hazard ratios for the association between elevated SII and key clinical outcomes in breast cancer patients.

The pooled hazard ratios for OS, DFS, and all-cause mortality consistently indicate that elevated SII is associated with adverse clinical outcomes in breast cancer. These associations are visually summarized in Figure 3, which presents a forest plot of the meta-analytic estimates and their corresponding confidence intervals.

## 4. Discussion

### 4.1. Interpretation of Key Findings: AI in Mammography

The synthesis of evidence from 2015 to 2025 demonstrates that AI in mammography has made substantial strides in improving breast cancer detection accuracy, reducing false positives and negatives, and significantly enhancing workflow efficiency [1,11]. AI algorithms, particularly deep learning models, have shown remarkable capabilities in autonomously identifying complex imaging patterns, thereby mitigating human subjectivity and inter-reader variability [2], especially in challenging cases such as dense breast tissue where conventional mammography’s sensitivity is limited [12,13]. The ability of AI to reduce radiologists’ reading time by 17–91% and triage examinations represents a tangible improvement in clinical workflow, potentially alleviating the burden on breast imaging specialists.

Crucially, emerging prospective trials are beginning to validate AI’s real-world efficacy, addressing the limitations inherent in the predominantly retrospective studies that characterized earlier research [14,15]. The interim results from the highly regarded MASAI randomized controlled trial, for instance, reported a 20% higher cancer detection rate in women whose mammograms were read by a radiologist using AI compared with those read by two radiologists without AI intervention [16]. Similarly, the AI-STREAM prospective multicenter cohort study demonstrated a significantly higher cancer detection rate (5.70‰ with AI-CAD vs. 5.01‰ without AI-CAD, *p* < 0.001) in a real-world, single-read setting, with no significant increase in recall rates [14]. This superior performance of AI-assisted reading, even when compared to the current “gold standard” of double-reading in some contexts, suggests a profound shift in how mammography screening could be conducted. This development has significant implications for optimizing screening programs globally, as it could reduce the reliance on extensive human resources (radiologists) and improve access to high-quality screening in underserved areas, thereby impacting global breast cancer mortality rates [17,18]. However, the current practice of some clinics charging patients an additional fee for AI analysis, which is often not covered by insurance, introduces a critical ethical and access barrier [19]. This financial imposition threatens to create a two-tiered system for advanced diagnostic services, where only those who can afford the extra cost benefit from AI’s advantages, potentially exacerbating healthcare disparities and undermining the broader public health goal of equitable access to effective screening [20]. Prior meta-analyses, including that by Alabousi et al. (2020) [19], demonstrated moderate diagnostic gains with AI but were limited by fewer reader studies and did not integrate recent prospective trials. Our findings build upon this by incorporating newer data from large-scale prospective evaluations, such as AI-STREAM and MASAI, which provide stronger evidence of AI’s utility in real-world screening settings. Additionally, while previous reviews often focused solely on model accuracy, our synthesis also emphasizes AI’s impact on workflow efficiency and its evolving role in triage strategies, offering a more implementation-oriented perspective [19]. These findings support the integration of AI as a second reader in organized screening programs, particularly in regions where double-reading is not feasible due to workforce limitations. AI tools could also serve in triage, prioritizing abnormal studies for expedited review. Pilot implementation in high-volume centers, accompanied by diagnostic performance auditing and radiologist feedback loops, would facilitate safe and scalable deployment. Until more robust prospective data become available, AI should complement rather than replace human interpretation in routine clinical practice.

### 4.2. Interpretation of Key Findings: SII as a Prognostic Factor

The review consistently demonstrates that a high SII is significantly associated with a poorer prognosis in breast cancer patients, as evidenced by its correlation with reduced OS, DFS, and lower rates of pCR after neoadjuvant chemotherapy [4,6,21]. This consistent prognostic value across various breast cancer subtypes and treatment contexts underscores the critical role of systemic inflammation in cancer progression. As a biomarker derived from routine peripheral blood counts, SII is cost-effective and easily accessible, making it an attractive tool for assessing the host’s immune-inflammatory state. Its ability to integrate the counts of neutrophils, platelets, and lymphocytes [22] offers a comprehensive reflection of the complex interplay between inflammation, coagulation, and immune activity, which are all implicated in tumorigenesis and progression [4,23].

While SII consistently predicts poorer outcomes, a notable challenge for its routine clinical implementation is the significant heterogeneity in optimal cut-off values reported across different studies [5,6]. This variability means that while SII is associated with prognosis, its precision as a standardized clinical tool for risk stratification is currently hampered. Clinicians cannot reliably apply a single threshold to classify patients across diverse populations or clinical settings. This limitation contrasts with more established prognostic factors like tumor stage or molecular subtypes, which benefit from clearer, widely adopted thresholds [10,24]. Despite this variability, the consistent association of a high SII with a higher tumor stage, triple-negative breast cancer, and poorer chemotherapy responses reinforces its clinical relevance. This suggests that SII, as an easily obtainable biomarker, could be integrated into existing risk stratification models to refine prognosis and guide treatment intensity, particularly for patients who might otherwise be categorized as lower risk based on traditional factors alone [25]. This highlights SII’s potential as a simple yet powerful indicator for identifying high-risk patients who may benefit from more aggressive or tailored therapies. The subgroup analyses explored variations in SII’s prognostic utility across molecular subtypes, treatment settings, and geographical regions. Due to the limited number of studies per subgroup and incomplete reporting, formal meta-regression could not be reliably conducted. Earlier meta-analyses, such as those by Yang et al. (2018) [5] and Cheng et al. (2024) [4], established SII as a significant prognostic marker across various cancers, including breast cancer. However, they often aggregated heterogeneous tumor types or lacked breast cancer-specific subgroup analyses. Our review expands this knowledge by focusing exclusively on breast cancer and including newer studies that evaluate SII in relation to neoadjuvant therapy outcomes and pCR. Moreover, we juxtapose SII against NLR and PLR using pooled metrics, thus reinforcing its superior discriminatory power in this specific oncologic context [4,5]. SII may be integrated as an adjunctive prognostic marker during initial patient evaluation, especially in settings where genomic profiling is not accessible. In triple-negative or HER2-positive subtypes, where treatment escalation decisions are often nuanced, SII may help flag patients at higher risk for relapse. Its simplicity and cost-effectiveness make it suitable for implementation in both tertiary centers and resource-limited environments, pending prospective validation and harmonization of threshold definitions.

### 4.3. Study Limitations

While this review builds upon prior meta-analyses by incorporating the most recent studies and emphasizing clinical implementation and biomarker comparison, several limitations must be acknowledged [26]. The adherence to PRISMA 2020 guidelines ensures a rigorous and transparent methodology, from protocol development to reporting. A comprehensive search strategy across multiple major electronic databases, complemented by hand-searching, aimed to minimize publication bias and capture a broad spectrum of relevant literature [27]. The dual independent review for study selection and data extraction, with a robust conflict resolution process, further enhances the objectivity and accuracy of the review [28]. Focusing on recent literature (2015–2025) ensures that the synthesized evidence reflects the most current advancements and clinical practices in both AI in mammography and SII as a prognostic factor.

However, several limitations inherent in the included primary studies, and consequently in this review, warrant consideration. For AI in mammography, a significant limitation is the predominance of retrospective studies in the current literature. While these studies demonstrate promising diagnostic accuracy, their retrospective nature limits the assessment of real-world efficacy and generalizability, as they are prone to selection bias and confounding. There are also ongoing ethical, medicolegal, and liability concerns that require careful consideration before widespread routine use of AI in breast imaging clinics. Furthermore, a lack of standardized validation across diverse populations, hardware, and clinical settings poses a challenge for broad applicability [15]. The emerging trend of charging patients out-of-pocket for AI analysis, which is not covered by insurance, introduces a critical access barrier and raises concerns about equitable healthcare provision.

For SII as a prognostic factor, a primary limitation is the considerable heterogeneity observed in optimal cut-off values for SII across different studies. This variability complicates the establishment of a universally applicable threshold for clinical decision-making. Additionally, there is variability in the specific patient populations and treatment regimens across studies, which can contribute to heterogeneity in pooled outcomes. While efforts were made to assess publication bias using statistical tests, the inherent nature of meta-analyses means that some degree of publication bias may still exist. General limitations of systematic reviews include their reliance on the quality and completeness of published data and the potential for reporting bias within primary studies [29].

### 4.4. Future Directions

The current state of research concerning both AI in mammography and SII underscores the pressing need for translational investigations aimed at bridging the gap between encouraging scientific findings and their application in routine clinical practice [14,15]. Although both approaches exhibit considerable promise, their widespread adoption remains contingent upon rigorous validation in real-world clinical environments, particularly across diverse patient populations. Furthermore, the development of clear protocols for their integration and interpretation is essential. This necessitates a transition from efficacy-focused research, typically conducted under controlled conditions, to effective research that assesses performance in everyday clinical settings.

In the domain of AI-assisted mammography, several priorities emerge. Chief among them is the urgent requirement for large-scale, prospective, randomized controlled trials designed to evaluate the technology’s real-world utility. Such studies should extend beyond diagnostic accuracy and encompass long-term outcomes, including mortality reduction, interval cancer detection rates, and cost-effectiveness [30]. Additionally, there is a need to investigate how AI can be optimally embedded within existing clinical workflows. This includes determining whether AI is best deployed as a first reader for low-risk cases, as a second reader providing a safety net for complex diagnoses, or as a triage tool to streamline caseloads. The impact of these integrations on radiologist performance and diagnostic confidence must also be systematically assessed.

Equally important is the formulation of comprehensive ethical guidelines and robust medicolegal frameworks to support the responsible use of AI in clinical practice [31]. This includes addressing issues of accountability, patient consent, and data security [32,33]. In parallel, standardized reimbursement policies must be established to ensure equitable access and to prevent the entrenchment of disparities in care. Another critical avenue of exploration involves the role of AI in developing personalized screening protocols tailored to individual risk profiles and breast density, which could ultimately refine screening intervals and modalities for greater efficacy.

Turning to SII, the research agenda should prioritize the standardization of its calculation methods, as well as the identification of optimal cut-off values that are both clinically meaningful and universally applicable [22,34,35]. Achieving this will likely require extensive prospective studies and may benefit from stratification according to breast cancer molecular subtypes, treatment stages, or patient-specific variables such as age and comorbidities [36]. Beyond standardization, validation of SII’s prognostic significance in specific subtypes of breast cancer, particularly when integrated with established molecular profiling techniques, is essential to determine its incremental value.

In addition, a deeper understanding of the biological pathways through which systemic inflammation, as captured by SII, contributes to tumor progression and influences treatment response is crucial [31]. This line of inquiry may help unlock new insights into cancer pathophysiology and inform the development of novel therapeutic approaches. Finally, SII’s potential to guide individualized treatment strategies merits close examination. By identifying patients who might benefit from more aggressive therapies or targeted anti-inflammatory interventions, SII could serve not only as a prognostic marker but also as a dynamic tool for therapeutic decision-making.

## 5. Conclusions

This meta-analysis and systematic review, covering literature from 2015 to 2025, synthesizes key advancements in breast cancer management through the integration of AI in mammographic screening and the prognostic application of SII. AI has demonstrated notable efficacy in enhancing diagnostic accuracy, particularly in complex scenarios such as dense breast tissue, where it often performs on par with or even exceeds that of human radiologists. Moreover, AI significantly improves workflow efficiency by decreasing interpretation time and facilitating examination triage, thus addressing persistent limitations in traditional screening methodologies.

Simultaneously, the review affirms that elevated SII levels correlate with poorer clinical outcomes in breast cancer, including diminished OS, reduced DFS, and lower rates of pCR after neoadjuvant chemotherapy. As SII is calculated from standard hematologic parameters, it stands out as a cost-effective and easily obtainable biomarker that reflects the role of systemic inflammation in tumor progression.

Taken together, the findings underscore the transformative potential of combining cutting-edge technologies with accessible biomarkers to advance breast cancer care. To translate these innovations into clinical practice effectively and equitably, it is imperative to undertake rigorous prospective trials evaluating AI’s real-world impact on long-term patient outcomes. Simultaneously, efforts should focus on developing ethical, legal, and reimbursement frameworks to support responsible AI implementation. For SII, standardization of calculation methods and cut-off values, alongside mechanistic research into its biological underpinnings, is essential. Through such multidisciplinary initiatives, the integration of AI and SII may enable more precise, individualized, and impactful approaches to breast cancer diagnosis and treatment worldwide.

## Figures and Tables

**Table 1:** (A): Characteristics of included prospective and retrospective studies on AI in mammography. (B): Characteristics of meta-analyses on AI in mammography.

**(A)**					
**Year**	**Study Design**	**Population (N)**	**AI Model/Algorithm**	**Comparator**	**Key Findings**
2025	Retrospective	578 images	CNN	Radiologist	Accuracy 88.5%, AUC 0.93, high specificity (92.7%), reduced false positives.
2025	Retrospective	434 mammograms	Lunit INSIGHT MMG	Radiologists	AI assistance significantly improved diagnostic performance for all readers (*p* < 0.05), especially for less experienced ones.
2024	Prospective	Screening mammograms	Two DL-based AI models	Radiologist	AUC 0.93 for both models; identified 81.8–82.5% of screen-detected cancers at threshold 1.
2025	Prospective	24,543 women	AI-CAD	Radiologist	CDR was significantly higher with AI-CAD (5.70‰ vs. 5.01‰, *p* < 0.001) in the single-read setting; no significant difference in RRs.
**(B)**					
**Year**	**Study Design**	**Population (N)**	**AI Model/Algorithm**	**Comparator**	**Key Findings**
2025	Meta-analysis	1,108,328 mammograms, 497,091 women	AI/ML algorithms	Radiologists	AI standalone AUCs were significantly higher than radiologists (0.87 vs. 0.81, *p* = 0.002) and reduced reading time (17–91%).

**Table 2:** Diagnostic performance metrics of AI models in mammography.

AI Application	Sensitivity (95% CI)	Specificity (95% CI)	AUC (95% CI)	Recall Rate	Cancer Detection Rate	Reading Time Reduction
Classification	-	92.7%	0.93	-	-	-
Detection	0.75 (0.65–0.83)	0.90 (0.82–0.95)	0.89 (0.84–0.98)	-	-	17–91%
Detection	-	-	0.93 (0.92, 0.94)	-	81.8–93.7% (Model A/B)	-
Detection	-	-	-	No significant difference	5.70‰ (with AI) vs. 5.01‰ (without AI)	-

**Table 3:** Comparison of SII with other inflammatory markers in breast cancer prognosis.

Inflammatory Marker	Outcome	Pooled HR/OR (95% CI) or AUC	Comparative Statement
SII	OS	AUC: 0.625	Superior to NLR (AUC 0.555) and PLR (AUC 0.571)
NLR	OS	HR: 1.78 (1.49–2.13)	Elevated NLR associated with poor prognosis
PLR	OS	HR: 1.32 (1.11–1.57)	Elevated PLR associated with worse prognosis
SII	Chemotherapy Response	AUC: 0.751	May eclipse NLR/PLR as a predictor

**Table 4:** Characteristics of included studies on SII as a prognostic factor.

Year	Study Design	Patient Population (N)	Breast Cancer Subtype Distribution	SII Cut-off Value Used	Key Prognostic Outcomes Assessed
2024	Meta-analysis	BC patients (pooled)	-	Varied	OS, DFS
2025	Cohort Study	21,058 females (557 BC)	-	SII/100: 5.09 (incidence), 5.22 (mortality)	Incidence, all-cause mortality
2024	Cohort Study	112 BC patients	-	598.5	pCR
2024	Cohort Study	1808 BC patients	HR-positive, HER2-negative, TNBC	586.40 (chemo response), 900 (relapse)	Chemotherapy response, Recurrence
2020	Cohort Study	249 BC patients	-	547	OS, pCR (comparison to NLR/PLR)
2024	Cohort Study	112 BC patients	-	598.5	pCR
2018	Meta-analysis	7657 cancer patients	Various cancers (incl. BC)	300–1600 (varied)	OS, TTR, PFS, CSS, DFS, RFS

**Table 5:** Pooled estimates of prognostic value for SII in breast cancer.

Outcome	Number of Studies	Number of Patients	Pooled HR/OR (95% CI)	Heterogeneity (I^2^, *p*-Value)
OS		-	HR: 1.97 (1.54, 2.52)	I^2^ = 76%
OS		7196	HR: 1.69 (1.42, 2.01)	-
DFS		-	HR: 2.07 (1.50, 2.86)	I^2^ = 79%
pCR		112	OR: (SII independent predictor)	-
All-cause mortality		557 (BC subset)	HR: 1.09 (1.04, 1.14) (above SII/100 = 5.22)	-

## Supplementary Materials

The following supporting information can be downloaded at: https://www.mdpi.com/article/10.3390/medicina61071170/s1, Table S1: List of Included Studies. Table S2: Studies on Systemic Immune-Inflammation Index (SII) in Breast Cancer (n = 7).

## Abbreviations

The following abbreviations are used in this manuscript:

AI	AI
SII	Systemic Immune-Inflammation Index
OS	Overall Survival
DFS	Disease-Free Survival
PFS	Progression-Free Survival
pCR	Pathological Complete Response
CNN	Convolutional Neural Network
DBT	Digital Breast Tomosynthesis
NLR	Neutrophil-to-Lymphocyte Ratio
PLR	Platelet-to-Lymphocyte Ratio
AUC	Area Under the Curve
PPV	Positive Predictive Value
NPV	Negative Predictive Value
CAD	Computer-Aided Detection
RCT	Randomized Controlled Trial
QUADAS-2	Quality Assessment of Diagnostic Accuracy Studies 2
NOS	Newcastle–Ottawa Scale
HR	Hazard Ratio
OR	Odds Ratio
CI	Confidence Interval
TTR	Time to Recurrence
CSS	Cancer-Specific Survival
RFS	Recurrence-Free Survival
