# Supplementary material for: Systematic Review and Meta-Analysis of AI-Assisted Mammography and the Systemic Immune-Inflammation Index in Breast Cancer: Diagnostic and Prognostic Perspectives
Source: Medicina (Kaunas). 2025 Jun 27;61(7):1170. doi: 10.3390/medicina61071170 (PMC12300038; doi:10.3390/medicina61071170)
Supplement: Supplementary file 1 [file medicina-61-01170-s001.zip › medicina-3687517-supplementary.pdf]

Supplementary Table: List of Included Studies

Table S1. Studies on AI in Mammography (n=5).

Title	URL
AI in 2D Mammography: Improving Breast Cancer Screening Accuracy	https://www.mdpi.com/1648-9144/61/5/809
The Future of Breast Cancer Organized Screening Program Through Artificial Intelligence: A Scoping Review	https://pmc.ncbi.nlm.nih.gov/articles/PMC11855082/
AI in 2D Mammography: Improving Breast Cancer Screening Accuracy	https://journals.plos.org/plosone/article?id=10.1371/journal.pone.0322925
Performance of Two Deep Learning-based AI Models for Breast Cancer Detection and Localization on Screening Mammograms from BreastScreen Norway	https://pubmed.ncbi.nlm.nih.gov/39907587
Diagnostic Accuracy of Breast Radiologists with and without AI-based Computer-	https://pmc.ncbi.nlm.nih.gov/articles/PMC11885569/

Aided Detection for Screening Mammograms in a Real-World, Single-Read Setting	

Table S2. Studies on Systemic Immune-Inflammation Index (SII) in Breast Cancer (n=7).

Title	URL
Prognostic role of the systemic immune-inflammation index and pan-immune inflammation value for outcomes of breast cancer: a systematic review and meta-analysis	https://www.europeanreview.org/article/34903
The association of systemic immune-inflammation index with incident breast cancer and all-cause mortality: evidence from a large population-based study	https://pmc.ncbi.nlm.nih.gov/articles/PMC11802490/
The role of systemic immune-inflammation index (SII) in complete pathological response (pCR) of breast cancer patients after neoadjuvant chemotherapy	https://pmc.ncbi.nlm.nih.gov/articles/PMC11564179/
Predictive significance of systemic immune-inflammation index in patients with breast cancer undergoing neoadjuvant chemotherapy	https://www.tandfonline.com/doi/full/10.2147/OTT.S434193
Systemic Immune-Inflammation Index Is Superior to Neutrophil to Lymphocyte Ratio in Prognostic Assessment	https://pmc.ncbi.nlm.nih.gov/articles/PMC7762645/

of Breast Cancer Patients Undergoing Neoadjuvant Chemotherapy	
Prognostic value of Systemic immune-inflammation index in cancer: A meta-analysis	https://pmc.ncbi.nlm.nih.gov/articles/PMC6160683/
Prognostic value of neutrophil-to-lymphocyte ratio and platelet-to-lymphocyte ratio for patients with breast cancer	https://pmc.ncbi.nlm.nih.gov/articles/PMC6675722/
